# Glycogene expression profiles based on microarray data from cervical carcinoma HeLa cells with partially silenced E6 and E7 HPV oncogenes

**DOI:** 10.1186/s13027-018-0197-2

**Published:** 2018-07-20

**Authors:** Miguel Aco-Tlachi, Ricardo Carreño-López, Patricia L. Martínez-Morales, Paola Maycotte, Adriana Aguilar-Lemarroy, Luis Felipe Jave-Suárez, Gerardo Santos-López, Julio Reyes-Leyva, Verónica Vallejo-Ruiz

**Affiliations:** 1 Centro de Investigación Biomédica de Oriente, Instituto Mexicano del Seguro Social, Km. 4.5 Carretera Federal Atlixco-Metepec, Atlixco, C.P. 74360 Puebla, Mexico; 2 Posgrado en Ciencias Microbiológicas, Benemérita Universidad Autónoma de Puebla, Edificio 103-J Cd. Universitaria, Col. San Manuel, C.P. 72570 Puebla, Pue Mexico; 3 Centro de Investigación Biomédica de Occidente, Instituto Mexicano del Seguro Social, Sierra Mojada 800, Col Independencia, C.P. 44340 Guadalajara, Jalisco Mexico; 4 CONACYT- Centro de Investigación Biomédica de Oriente, Instituto Mexicano del Seguro Social, Km. 4.5 Carretera Federal Atlixco-Metepec, Atlixco, C.P. 74360 Puebla, Mexico

**Keywords:** Microarrays, Glycogene, Cervical cancer, HeLa cells, Human papillomavirus, E6 oncoprotein, E7 oncoprotein

## Abstract

**Background:**

Aberrant glycosylation is a characteristic of tumour cells. The expression of certain glycan structures has been associated with poor prognosis. In cervical carcinoma, changes in the expression levels of some glycogenes have been associated with lymph invasion. Human papillomavirus (HPV) infection is one of the most important factors underlying the development of cervical cancer. The HPV oncoproteins E6 and E7 have been implicated in cervical carcinogenesis and can modify the host gene expression profile. The roles of these oncoproteins in glycosylation changes have not been previously reported.

**Methods:**

To determine the effect of the E6 and E7 oncoproteins on glycogene expression we partially silenced the E6 and E7 oncogenes in HeLa cells, we performed a microarray expression assay to identify altered glycogenes and quantified the mRNA levels of glycogenes by RT-qPCR. A protein-protein interaction network was constructed to identify potentially altered glycosylation pathways.

**Results:**

The microarray analysis showed 9 glycogenes that were upregulated and 7 glycogenes that were downregulated in HeLa shE6/E7 cells. Some of these genes participate in glycosylation related to Notch proteins and O-glycans antigens.

**Conclusions:**

Our results support that E6 and E7 oncoproteins could modify glycogene expression the products of which participate in the synthesis of structures implicated in proliferation, adhesion and apoptosis.

## Background

Glycosylation changes have been reported in cancer, and glycan structures are found in secreted proteins, membrane glycoproteins, and glycolipids. Glycans are involved in cellular adhesion, tumour proliferation, apoptosis, invasion, metastasis, angiogenesis and signal transduction [1].

In cervical cancer, the increased expression of some glycogenes such as *ST6GAL1* and *ST3GAL3*, has been correlated with deep stromal invasion and lymph node metastasis [2]. The increased expression of sialyltransferases genes could be related to an overexpression of sialylated antigens such as sialyl-T, sialyl-Le(a), and sialyl-Le(x), identified in cervical neoplasia [3–5].

Polylactosamine (polyLacNAc) is a glycan structure expressed during development and carcinogenesis, and β1,3-N-acetylglucosaminyl transferases (β3GnTs) participate in its synthesis. In cervical tissue, the increased expression of β3GnT2 has been detected in cervical intraepithelial neoplasia 3 (CIN3), and polyLacNAc expression is higher in cancer tissue [6]. The gene regulation of glycogenes is very complex, and little information exists about the factors that modify their expression during cervical transformation.

Cervical cancer tumours are associated with high-risk human papillomavirus infections. HPV-16 is the most prevalent high-risk HPV type, followed by HPV-18 and HPV-31 [7]. The HPV-18 type is the most prevalent genotype in cervical adenocarcinoma [8]. One of the key events of HPV-induced cervical cancer is the integration of the HPV genome into the host chromosome [9]. Expression of E6 and E7 genes is necessary for cell transformation induction and contributes to genome instability [10]. These viral oncoproteins have been implicated in the altered expression of different genes via different mechanisms. It has been reported that E6 can increase the expression of hTERT, the catalytic domain of telomerase, via transcription factor interactions, transcription repressor degradation, and chromatin structure modifications [11–13]. There are no reports about the roles of the E6 and E7 oncoproteins in glycosylation changes in the cervix.

The objective of this work was to identify glycogenes that modify their expression by partially silencing the HPV-18 E6 and E7 oncogenes in HeLa cells using microarray analysis.

## Methods

### Cell culture

HeLa cell line (VPH18+) from cervical cancer was used to perform the gene silencing. Cells were cultured and maintained in Dulbecco’s Modified Eagle’s Medium (DMEM) containing Earle’s salts and L-glutamine (DMEM; Sigma, St. Louis, MO, USA), and supplemented with 10% foetal bovine serum, and 100 μg/ml streptomycin (Sigma). Cells were maintained at 37 °C in a 5% CO2 atmosphere. The culture medium was replaced every two days. Subconfluent adherent cells were harvested using a mixture of trypsin (0.025%) and EDTA (0.02%; Sigma) and washed with phosphate-buffered saline.

### Gene silencing and clonal selection

Gene silencing in HeLa parental cells was achieved by cloning the shRNA sequence 5’-CTAACACTGGGTTATACAA-3′ into the pLVX-sh vector (pLVX-shE6/E7) (Clontech Laboratories, Mountain View, CA, USA). The 19-nucleotide sequence targets the E6 and E7 bicistronic mRNA [14]. As a control, the sequence 5’-GACTTCATAAGGCGCATGC-3′ [15] was cloned into the pLVX-sh vector (pLVX-shControl). To obtain recombinant lentiviral particles carrying the respective constructs, Lenti-X 293 T cells were co-transfected with either pLVX-shControl or pLVX-shE6/E7 and Lenti-X HTX Packaging Mix (Clontech Laboratories, Mountain View, CA, USA). Lentiviruses were harvested from the supernatant and filtered. The presence of viral particles in the filtrate was confirmed using the LentiX-Gostik kit (Clontech Laboratories, Mountain View, CA, USA). Supernatants bearing lentivirus pLVX-shControl or pLVX-shE6/E7 were then used to transduce HeLa cells. Transduced cells were selected with puromycin and when the culture reached 60% confluence, the cells were trypsinized and diluted to obtain monoclonal cultures. Screening of several HeLa clones carrying either shcontrol or shE6/E7 was performed by RT-qPCR to select clones with no change in the expression levels of E6/E7 or the best degree of gene silencing compare with parental HeLa cells (data not shown).

### RT-qPCR

Total RNA from shcontrol and shE6/E7 HeLa monocultures was obtained using the NucleoSpin RNA II kit (Macherey-Nagel, Düren, Germany). In total, 500 ng of RNA was used to synthesize cDNA using random primers and the RevertAid First-Strand cDNA Synthesis kit (Thermo Fisher Scientific, USA).

To show that the amplification efficiencies of the E6, E7, *POFUT1*, *XXYLT1*, *DPY19L1, ALG14, UGT8*, *PIGV*, and *GALNT1* genes were optimal, standard curves were constructed with the following concentrations: 10, 1, 0.1, 0.01, and 0.001 ng/μL; HPRT was used as an endogenous gene. Each reaction was performed in a final volume of 10 μL comprising the following: 1 μL of cDNA template, 5 μL of 2X Maxima SYBER Green/Rox qPCR Master Mix (Thermo Fisher Scientific, USA), and 0.5 μL of 10 mM forward and reverse primers for the E6, E7, *POFUT1, XXYLT1*, *DPY19L1*, *ALG14, UGT8*, *PIGV, GALNT1* and HPRT genes (Table 1). The reactions were performed with a StepOne Real-Time PCR System (Applied Biosystems, Foster, CA), and the conditions were as follows: 95 °C for 10 min, followed by 40 cycles of 95 °C for 30 s, 60 °C 30 s, and 70 °C for 30 s.

**Table 1:** Sequences of the oligonucleotides used in the RT-qPCR assays to quantify E6, E7 and glycogenes expression level

Name	Sequence	PCR Product
E6 Forward	5’GCGACCCTACAAGCTACCTGAT 3´	295 bp
E6 Reverse	5’GCACCGCAGGCACCTTAT 3´	
E7 Forward	5´ TGTCACGAGCAATTAAGCGACT 3´	215 bp
E7 Reverse	5´ CACACAAAGGACAGGGTGTTC 3’	
POFUT1 Forward	5’CAGCCCAGTTCCCCGTCCTA3´	190 bp
POFUT1 Reverse	5’GAGCCTGCAGTCCCGTCCTTC3´	
UGT8 Forward	5’AAACCAGCCAGCCCACTACCAG3´	93 bp
UGT8 Reverse	5’GACACCAGCTCCAAAAGACACCAA3´	
XXLYLT1 Forward	5’GTGCTGGCTTGGGAACCTACTA3´	230 bp
XXLYLT1 Reverse	5’GCGGAACTGCCAGAATGTGT3´	
DPY19L1 Forward	5’GAGAGTGTACCCGTGTAATGTG3´	134 bp
DPY19L1 Reverse	5’GAGTGCAATCAAGCTTCCTCTA3´	
PIGV Forward	5’ CCTGGGCAACTTGGACATA 3’	95 bp
PIGV Reverse	5’ GGGCTTCTCTAGGGTCTTATTG 3’	
ALG14 Forward	5’ CCGGGAGTCTCTCAGTATCTT 3’	100 bp
ALG14 Reverse	5’ TCTAGGTGAGTAGGCATTGGA 3’	
GALNT1 Forward	5’ GGATAAAGCCACAGAAGAGGATAG 3’	94 bp
GALNT1 Reverse	5’ CAGGGTGACGTTTCGAAGAA 3’	
HPRT Forward	5’CCTGGCGTCGTGATTAGTGATGAT3´	136 bp
HPRT Reverse	5’CGAGCAAGACGTTCAGTCCTGTC3´	

To determine the mRNA levels the final reaction volume of 10 μL included 1 μL of cDNA template (0.5 ng/μL final concentration for the E6 and E7 genes and 0.1 ng/μL for the glycogenes), 5 μL of 2X Maxima SYBR Green/Rox qPCR Master Mix (Thermo Scientific, California, USA), 0.5 μL of forward and reverse primers (0.5 μM final concentration) and 3 μL of RNase free water. RT-qPCR was performed under the following conditions: 95 °C for 10 min, followed by 40 cycles of 95 °C for 30 s, 60 °C for 30 s and 70 °C for 30 s. The transcript levels of E6, E7 and the glycogenes were normalized to those of HPRT.

Relative quantification was performed using the comparative CT method with the formula 2^-ΔΔCT^. The qPCR reaction was performed using the StepOneReal-Time PCR System (Applied Biosytems, Foster, CA).

### Western blot

Protein extraction was performed by mechanical lysis from non-treated, shcontrol and shE6/E7 monocultures at 90% confluence. Next, 50 μg protein samples of each total cell extract were separated by 12% SDS-PAGE to analyze p53, whereas 70 μg protein samples were separated by 15% SDS-PAGE to analyze E7. Samples were transferred to a polyvinylidene fluoride Immobilon*-*P membrane (Millipore, Darmstadt, Germany) and probed overnight at 4 °C with the antibodies anti-p53 (1:1000; Abcam ab1101), anti-E7 (1:1000; Abcam ab100953), and anti-β-actin (1:3000; Abcam ab8224). Signals were detected with an anti-HRP-conjugated secondary antibody (1:4000; Abcam ab97046) and developed with the ImmPACT™ DAB peroxidase substrate (Vector, Burlingame, CA. USA). Densitometric analysis was performed with ImageJ software.

### Hybridization and microarray

Microarray analysis was performed at the Cellular Physiology Institute of UNAM as follows: total RNA from cell cultures was extracted with a Direct-zol™ RNA MiniPrep kit (Zymo Research, Irvine, CA, USA). Next, 10 μg of total RNA was used for cDNA synthesis incorporating dUTP-Alexa555 or dUTP-Alexa647 employing the First-Strand cDNA labelling kit (Invitrogen). Incorporation of the fluorophore was analyzed using the absorbance at 555 nm for Alexa555 (HeLa shE6/E7) and 650 nm for Alexa647 (HeLa shControl). Equal quantities of labelled cDNA were hybridized using UniHyb hybridization solution (TeleChem International INC) with a chip containing 35 K genes. Acquisition and quantification of array images were performed using the ScanArray 4000 instrument with its accompanying software ScanArray 4000 from Packard BioChips, USA. For each spot, the Alexa555 and Alexa647 density and background mean values were calculated with ArrayPro Analyzer software from Media Cybernetics. Microarray data analysis was performed with GenArise (free) software, developed in the Computing Unit of the Cellular Physiology Institute of UNAM (http://www.ifc.unam.mx/genarise/). GenArise carries out several transformations: background correction, normalization, intensity filter, replicates analysis and selection of differentially expressed genes. Analyzed data were submitted to the NCBI-Gene Expression Omnibus database (accession number GSE90930).

### Analysis of biological processes

Altered genes were analyzed using DAVID (Database for Annotation, Visualization, and Integrated Discovery) software (http://david.abcc.ncifcrf.gov/). Based on gene ontology (GO) [16], genes were classified according to their function in a biological process (BP), and a *p*-value < 0.05 was considered statistically significant.

### Identification of glycogenes

We identified 336 glycogenes reported to date using the GlycoGene database (https://acgg.asia/ggdb2/index?doc), Consortium for Functional Glycomics-CAZy database (http://www.cazy.org/) and the published reports of glycogenes not included in the databases: *DPY19L1* gene, [17] and the *MANBAL* gene [18].

We selected genes with altered expression in the microarray corresponding to glycogenes.

### Protein-protein interaction network

Predicted interaction network analysis was performed with Cytoscape 3.4.0 and the ‘StringWSClient’ plugin, considering a score of 0.4 as the confidence level.

### Statistical analysis

Statistical analysis of the RT-qPCR results was performed using the Graph Pad programme. A One-way analysis of variance followed by Tukey’s post-test was performed. A *p*-value < 0.05 was considered statistically significant.

## Results

### E6 and E7 oncogenes expression by RT-qPCR

**Fig. 1:**
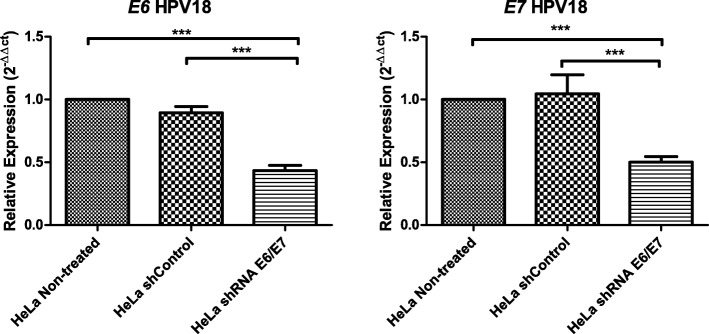
Inhibition of HPV18 E6/E7 mRNA expression by shRNA E6/E7 in a HeLa cell line. HeLa cells were transfected with shRNA or shcontrol, and the relative expression of E6/E7 was determined by real-time PCR. The x-axis shows the different experiment groups; the y-axis shows the relative E6 and E7 mRNA expression, which was normalized to that in non-treated HeLa cells. Three independent experiments carried out in triplicate are shown, and the groups were analyzed by one-way analysis of variance (ANOVA) with the Tukey test. ***p < 0.001

RT-qPCR was performed to quantify and compare the E6 and E7 mRNA expression levels in HeLa-non-treated, HeLa shcontrol and HeLa-shE6/E7 cells. The E6 mRNA expression level in HeLa shcontrol cells was like that in HeLa-non-treated cells. By contrast, E6 mRNA was decreased by 60% in HeLa shE6/E7 cells compared to that in HeLa-non-treated and HeLa shcontrol cells (*p* < 00.1) (Fig. 1). Similar results were obtained after analyzing E7 mRNA levels; its expression was decreased by 50% in HeLa shE6/E7 cells compared to that in HeLa-non-treated and HeLa shcontrol cells (*p* < 00.1).

### E6 and E7 proteins expression by western blot analysis

**Fig. 2:**
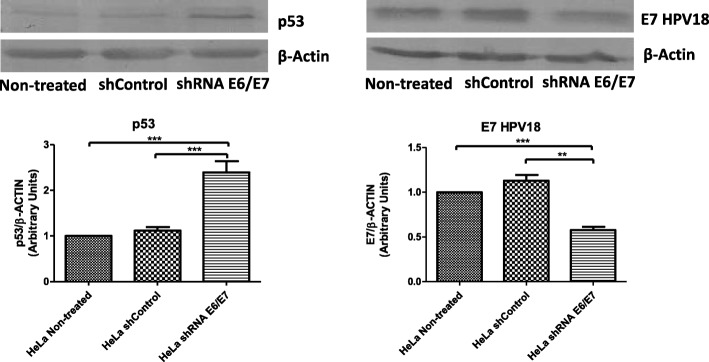
Evaluation of p53, E7 and β-actin protein expression in a HeLa cell line. Extracts from HeLa non-treated cells, stably-transduced HeLa shcontrol cells and HeLa shE6/E7 cells were analyzed by Western blotting. The derived histograms show that the p53 protein expression was up-regulated, while E7 expression was down-regulated in HeLa shE6/E7 cells, compared to that in HeLa non-treated and HeLa shcontrol cells. Protein levels were normalized to those of β-actin. Scanning densitometry was used to quantify images for the bar graphs. Three independent experiments carried out in triplicate are shown and the groups were analyzed by one-way analysis of variance (ANOVA) with the Tukey test ***p < 0.001, **p < 0.05

We analyzed the p53 and E7 protein expression levels by Western blot. First, we indirectly analyzed the expression of the E6 protein by evaluating the quantity of the p53 protein, since the oncoprotein induces p53 degradation and can thus serve as a marker of E6 expression. The latter was analyzed due to reports that E6 detection is difficult using Western blot in the HeLa cell line [19]. The p53 protein level was increased in HeLa-shE6/E7 cells by 1.5- fold compared to that in HeLa-non-treated and HeLa shcontrol cells (*p* < 0.001) (Fig. 2), suggesting a decrease in E6 protein expression. By contrast, we analyzed E7 expression by detecting the protein directly. As expected, E7 protein expression was decreased by 0.5-fold in HeLa-shE6/E7 cells compared to that in HeLa-non-treated (*p* < 0.001) and HeLa shcontrol cells (*p* < 0.05) (Fig. 2). In summary, these results show that HeLa shE6/E7 cells display downregulated E6 and E7 mRNA and protein expression.

### Cell organization, signalling and adhesion are the most affected biological processes under E6 and E7 downregulation

To analyze genes under E6 and E7 regulation, we performed a complete genome microarray comparing transcripts within HeLa shE6/E7 cells and HeLa shcontrol cells. Using GeneArise software, we identified 1157 genes exhibiting altered expression profiles in HeLa shE6/E7 cells compared with the control. The results showed altered genes with a Z-score > 2 for upregulated genes and a Z-score < 2 for downregulated genes. Thus, we identified 544 upregulated genes and 613 downregulated genes in E6 and E7 compared with the control (accession number GSE90930).

**Fig. 3:**
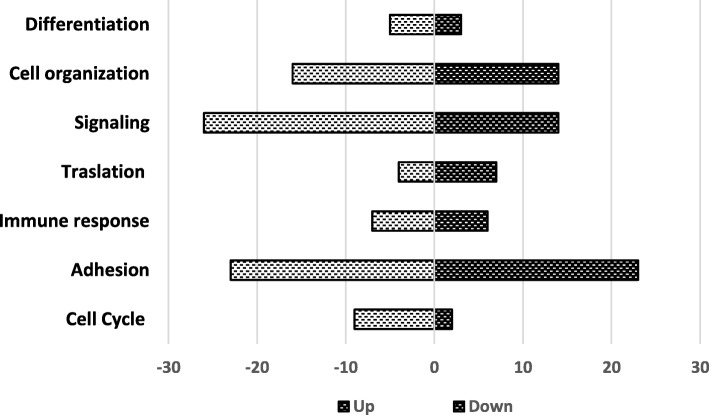
Gene ontology-based biological process terms. Genes that were differentially expressed by the partial silencing of HPV18 E6 and E7 were grouped according to their biological processes. Both up- and downregulated genes are shown

With the aim of been able to associate the 1157 altered genes according to their function, we performed an analysis with DAVID software and GO. The 1157 altered genes were related to differentiation, cell organization, signalling, translation, immune response, adhesion and cell cycle. Importantly, the results show that most of the expression changes in HeLa shE6/E7 cells correspond to genes associated with cell organization, signalling and cell adhesion (Fig. 3).

### Glycogene expression is altered by the E6 and E7 oncoproteins

Next, to identify glycogenes among the altered genes in the microarray, we first identified all the glycogenes reported to date, by searching the GlycoGene database, Consortium for Functional Glycomics-CAZy database and published reports. Among the 336 glycogenes included in the database, 9 genes were upregulated in HeLa shE6/E7 cells (Z score > 2), including *ALG14*, *POFUT1, FUT4, MAN2A1, DPY19L1, C3orf21* (*XXYLT1*), *UGT2B17, IDUA* and *UGCGL1.* A total of 7 glycogenes were downregulated (Z score < 2), including *GALNT1*, *B4GALT2*, *UGT8*, *MANBAL*, *PIGV*, *FCMD* and *C1GALT1C1*.

### Glycogene expression by qRT-PCR

**Fig. 4:**
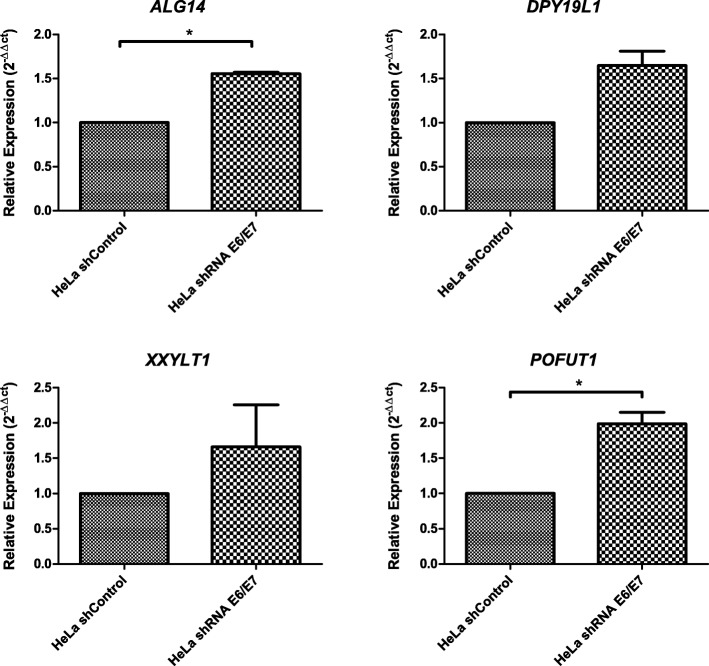
Expression of *POFUT1*, *DPY19L1*, *XXYLT1*, and *ALG14* in shcontrol and shE6/E7 HeLa cells. The mRNA expression levels were determined by RT-qPCR. The mean ± SD of three independent experiments carried out in triplicate assays is shown; *P* < 0.05

Among the 16 glycogenes altered in the microarray assay, we evaluated 7 by RT-qPCR. The mRNA levels were evaluated for *POFUT1*, *DPY19L1*, *XXYLT1*, *ALG14*, *UGT8*, *PIGV*, and *GALNT1* in HeLa shcontrol and HeLa-shE6/E7 cells. The *POFUT1* and *ALG14* mRNA levels were increased on HeLa shE6/E7 cells compared to those in HeLa shcontrol cells (*p* < 0.05) (Fig. 4). *DPY19L1* and *XXLT1* showed increased mRNA levels in HeLa shE6/E7 cells, but they were not statistically significant (Fig. 4).

**Fig. 5:**
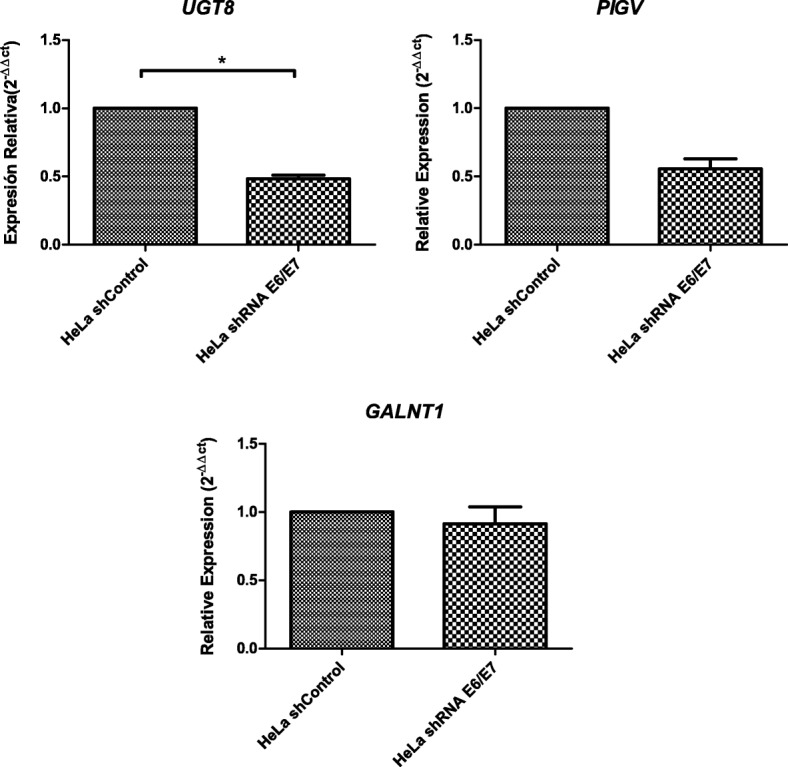
Expression of *UGT8*, *PIGV*, and *GALNT1* in shcontrol and shE6/E7 HeLa cells. The mRNA expression levels were determined by RT-qPCR. The mean ± SD of three independent experiments carried out in triplicate is shown; *P* < 0.05

The glycogenes downregulated in the microarray, *UGT8*, *PIGV*, and *GALNT1* were evaluated by RT-qPCR. Only *UGT8* showed statistically significant decreased expression in HeLa shE6/E7 cells; *PIGV* showed decreased expression but it was not statistically significant, and *GALNT1* did not showed expression changes (Fig. 5).

Next, we determined whether the glycogenes with altered expression in the microarray have been previously reported to have modified expression in cancerous tissues. Among the 9 upregulated genes, only *POFUT1, FUT4, UGT2B17,* and *MAN2A1* have been reported to be altered in cancerous tissues. *FUT4* is increased in leukaemia, gastric, breast and colorectal cancer; *UGT2B17* is increased in endometrial cancer; *POFUT1* is increased in glioblastomas and oral squamous cell carcinoma*,* and *MAN2A1 is* decreased in glioblastoma (Table 2).

**Table 2:** Glycogenes upregulated in HeLa shE6/E7 cells

Gene/enzyme	Enzyme function	Gene alteration in cancer and oncogenic associated-effects
*ALG14* UDP-N-Acetylglucosaminyltransferase Subunit	Associates with ALG13 and transfers a GlcNAc on GlcNAc-PP-Dol (second step of N-linked glycosylation)	Not reported
*FUT4* Fucosyltransferase 4	Catalyzes the transfer of fucose (Fuc) residues from GDP-Fuc to [Fucα1 → 2Galβ1 → 4GlcNAcβ1 → R] in α-1, 3 linkage	mRNA upregulated in acute myeloid leukaemia [54], gastric [30] and colorectal cancers [32]. Protein upregulated in breast cancer was associated with proliferation and metastasis [31]
*MAN2A1* Mannosidase Alpha Class 2A Member 1	Hydrolyzes two peripheral mannosyl residues from Manαl--6(Manαl--3) Manαl--6(GlcNAcβ1--2Manα1--3) [Manβ1--4GlcNAcβ1--4GlcNAcβ1]- asparagine structure	MAN2A1– FER tyrosine kinase fusion gene is expressed in liver tumours, oesophageal adenocarcinoma, glioblastoma multiforme, prostate tumours, non-small cell lung tumours, and ovarian tumours [55].
*DPY19L1* Dpy19 like 1 (*C. elegans*)	Participates in the C-mannosylation of tryptophan residues on target proteins.	Not reported
*C3orf21 (XXYLT1)* Xyloside Xylosyltransferase 1	Elongates the O-linked xylose-glucose disaccharide attached to EGF-like repeats (Notch proteins) by catalyzing the addition of the second xylose	Lower frequency of wild type genotype in the C3orf21 gene rs 2,131,877 locus in lung adenocarcinoma tissues [56]
*IDUA* Iduronidase, Alpha-L-	Cleaves α-linked iduronic acid residues from the nonreducing end of the glycosaminoglycans (GAGs), heparan sulfate, and dermatan sulfate	Not reported
*UGT2B17* UDP Glucuronosyltransferase Family 2 Member B17	Catalyzes the glucuronidation of steroids (detoxification)	mRNA upregulated in endometrial cancer [33]
*POFUT1* Protein O-Fucosyltransferase 1	Catalyzes the reaction that attaches fucose through an O-glycosidic linkage to a conserved serine or threonine of EGF domains	mRNA upregulated in glioblastomas [26] Protein upregulated in oral squamous cell carcinoma [29]
*UGCGL1* UDP-Glucose Glycoprotein Glucosyltransferase 1	Reglucosylates single N-glycans near the misfolded part of the protein	Not reported

Table showing upregulated glycogenes, the names of the coded enzymes, their alteration in different cancer types and their oncogenic-associated-effects

Next, we compared the downregulated glycogenes in HeLa shE6/E7 cells with those reported in cancerous tissues. Only 4 glycogenes have been reported in cancer. *GALNT1* is increased in bladder cancer, *C1GALT1C1* is increased in colorectal cancer and *UGT8* is increased in breast cancer. For *B4GALT2* missense mutations, have been reported in colon cancer. The remaining glycogenes have not been reported to be altered in cancerous tissues (Table 3).

**Table 3:** Glycogenes downregulated in HeLa shE6/E7 cells

Gene/enzyme	Enzyme function	Gene alteration in cancer and oncogenic- associated-effects
*PIGV* Phosphatidylinositol Glycan Anchor Biosynthesis Class V	Transfers the second mannose in the glycosylphosphatidylinositol (GPI) anchor	Not reported
*GALNT1* Polypeptide N-Acetylgalactosaminyltransferase 1	Transfers N-acetylgalactosamine (GalNAc) to a serine or threonine residue O-glycosylation	mRNA upregulated in bladder cancer stem cells [57]
*C1GALT1C1* C1GALT1 Specific Chaperone 1	Specific chaperone assisting the folding/stability of C1GALT1, for the generation of core 1 O-glycan T antigen	mRNA and protein upregulated in colorectal cancer [27]
*FCMD* Fukutin	Glycosyltransferase involved in the biosynthesis of α-dystroglycan (α-DG)	Not reported
*MANBAL* Mannosidase, beta A, lysosomal-like	Mannosidase beta-like	Not reported
*B4GALT2* Beta-1,4-Galactosyltransferase 2	Transfers galactose to the terminal N-acetylglucosamine of complex-type N-glycans	Missense mutations in B4GALT2 gene in colon cancer with a predictive deleterious phenotype [58]
*UGT8* Ceramide UDP-Galactosyltransferase	Catalyzes the transfer of galactose to ceramide (biosynthesis of GalCer)	mRNA and protein upregulated in breast [28] and increased protein expression in lung cancer [59]

Table showing downregulated glycogenes, the names of the coded enzymes and their alteration in different cancer types

### E6 and E7 activity may downregulate the notch glycosylation pathway and upregulate Tn and T antigens synthesis

**Fig. 6:**
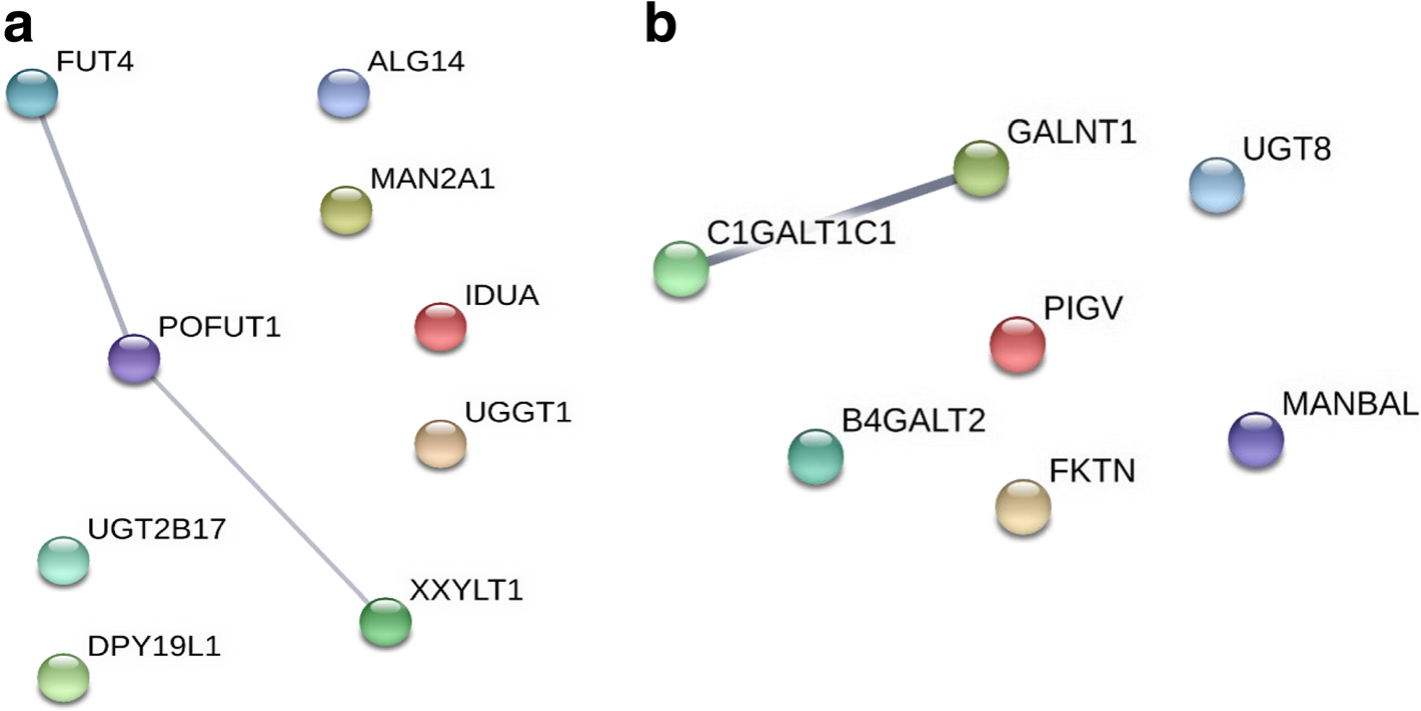
The protein-protein interaction network built in STRING for the glycogenes altered by the partial silencing of E6 and E7. The glycogenes were loaded in a list, and the networks upregulated (**a**) and downregulated (**b**) glycogenes are shown. The thickness of the line refers to the level of interaction. The thickest line refers to a stronger interaction

To identify possible functional association among the enzymes coded by the altered glycogenes under E6 and E7 downregulation, we examined data with Cytoscape software to generate predicted protein-protein interactions. Analyses among all upregulated glycogenes revealed a network whithin 3 glycogenes: *FUT4, POFUT1, and XXYLT1* (Fig. 6a). By contrast, analyses among downregulated genes showed an interaction only between *GALNT1* and *C1GALT1C1* (Fig. 6b). These results suggest that *FUT4, POFUT1 and XXYLT1* as well as *GALNT1* and *C1GALT1C1* could be participating in the same glycosylation pathway.

**Fig. 7:**
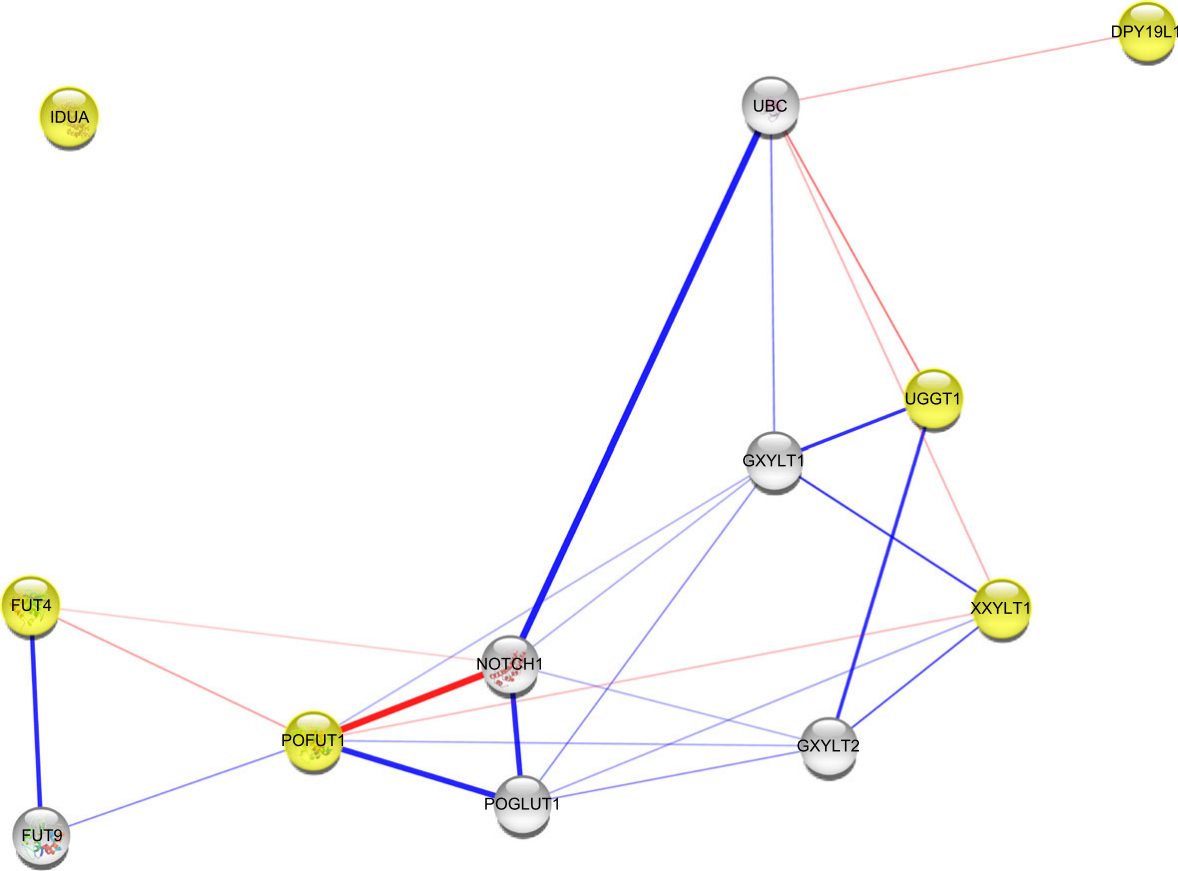
The protein-protein interaction network built in Cytoscape for the altered glycogenes and five proteins with which they could interact. The glycogenes were loaded in a list, and the network of glycogenes upregulated in HeLa cells with partially silenced E6/E7 genes is shown. Glycogenes are shown in yellow, and the thickness of the line refers to the level of interaction. The thickest line refers to a stronger interaction. The red line refers to the interaction of the glycogene and the target protein to be glycosylated

Next, with the aim of investigating potential targets of the up- and downregulated glycogenes, an analysis was performed by text mining when considering 5 more proteins that could be interacting with the altered glycogenes. Thus, the network among upregulated glycogenes revealed a direct interaction between *NOTCH1* and *POFUT1* (Fig. 7).

**Fig. 8:**
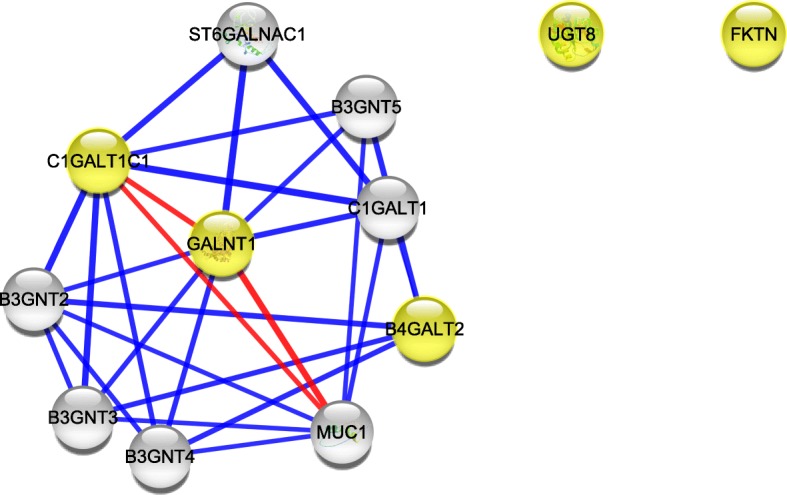
The protein-protein interaction network built in Cytoscape for the altered glycogenes and five proteins with which they could interact. The glycogenes were loaded in a list, and the network of glycogenes downregulated in partially silenced HeLa cells is shown. Glycogenes are shown in yellow, and the thickness of the line refers to the level of interaction. The thickest line refers to a stronger interaction. The red line refers to the interaction of the glycogene and the target protein to be glycosylated

Analysis among the downregulated genes revealed a direct interaction within *GALNT1*, *C1GALT1C1* and *MUC1* (Fig. 8), a protein that carries the Tn antigen, which is present in most of the cancers, including uterine cervical cancer cells [20–22].

## Discussion

Glycosylation changes in cancer have been associated with immune modulation, cell-matrix interactions, cell invasion, metastasis, and angiogenesis [23], and some of these changes could be due to the dysregulation of glycogenes at the transcriptional level (25–28). In cervical neoplasia, an increased expression of the sialyltransferase genes *ST6GAL1* and *ST3GAL3* has been reported as well as increased sialic acid and the tumour antigens sLe(X), Tn and sTn [2, 4, 5, 24, 25]. The role of HPV infection in the glycosylation changes in cervical neoplasia has not been studied. The viral oncoproteins E6 and E7 play a role in cellular transformation and have been implicated in the altered expression of several genes. Notably, the E6 and E7 oncoproteins can modulate gene expression by different mechanisms. It has been reported that E6 can increase the expression of hTERT via transcription factor interactions, transcription repressor degradation, and chromatin structure modifications [11–13]. The role of viral oncoproteins in the glycosylation changes detected in cervical cancer could be due to changes in glycogene expression.

In the present study, we report the potential effects of the E6 and E7 oncogenes in the expression changes of glycogenes. Analysis of the microarray assay results of HeLa cells with partially silenced E6 and E7 oncogenes showed that the cells displayed altered expression of the glycogenes *ALG14*, *POFUT1, FUT4, MAN2A1, DPY19L1, XXYLT1*, *UGT2B17, IDUA, UGCGL1, GALNT1, B4GALT2, UGT8, MANBAL, PIGV, FCMD,* and *C1GALT1C1*. Specifically,the results suggest that the E6 and E7 oncoproteins upregulate the expression of of *GALNT1, B4GALT2, UGT8, MANBAL, PIGV, FCMD,* and *C1GALT1C1* whereas they downregulate *ALG14*, *POFUT1, FUT4, MAN2A1, DPY19L1, XXYLT1*, *UGT2B17, IDUA,* and *UGCGL1.* By RT-qPCR, we confirmed the downregulation of UGT8 and the upregulation of *POFUT1* and *ALG14* in HeLa shE6/E7 cells. We observed that the mRNA level changes were not statistically significant for *XXYLT1*, *DPY19L1, and PIGV.* Although these glycogenes have not yet reported to be altered in cervical cancer, some have been reported to be aberrantly expressed in other cancer types, suggesting that they could be implicated in cervical transformation. In this manner, *MAN2A1* has been reported to be altered in glioblastoma [26], *GALNT1* is altered in bladder cancer, *C1GALT1C1* is altered in colorectal cancer [27], and *UGT8* is altered in breast cancer [28]. By contrast, our results indicate that *POFUT1*, *FUT4,* and *UGT2B17* are upregulated when E6 and E7 are knocked down, suggesting that the expression of these viral oncoproteins downregulates their expression. These results are contrary to reports on other cancer types, such as in leukaemia, glioblastoma, oral squamous cell carcinoma, gastric cancer, breast cancer, colorectal cancer, and endometrial cancer, wherein these genes are upregulated [26, 29–33]. These results indicate that the underlying mechanisms involved in their regulation are different depending on the type of cancer; for example, in human renal cell carcinoma, downregulation of *ST3GAL4* is associated with malignant progression, while in gastric cancer, upregulation is associated with malignant behaviour [34, 35].

The Human Protein Atlas database shows that the protein expression levels of Fut4, Man2A1 and Ugt2b17 are not increased in samples from patients with cervical cancer, and POFUT1 is detected at low and medium expression levels; these results agree with our results suggesting that the respective glycogenes are down-regulated in the presence of the E6/E7 oncoproteins. Moreover, *GALNT1,* and *C1CAGLT1C*, with higher expression in HeLa control cells, are reported to have medium and high expression levels in cervical cancer samples in this database, suggesting that viral oncoproteins could increase their expression in cervical cancer. Remarkably, in general, the data point to the same glycogene expression profile in cervical cancer.

In addition, functional bioinformatic analysis allowed us to elucidate the possible altered biosynthetic pathway considering the altered glycogenes and their potential targets. Thus, we identified that under higher E6 and E7 expression, the components of the Notch glycosylation pathway are downregulated, whereas the components of the synthesis of Tn and T antigens are upregulated. Interestingly, both mechanisms are aberrantly regulated in cancerous cell phenotypes [20, 36–38].

In the first case, analysis of the downregulated glycogenes under E6 and E7 activity and their potential targets showed a direct interaction within Pofut4 and Notch1. Pofut1 is an O-fucosyltranferase that modifies the extracellular EGF-like domains of Notch transmembrane proteins. The enzyme attaches a fucose via an O-glycosidic linkage to a conserved serine or threonine for proper protein folding [39]. Interestingly, defects in Notch receptor fucosylation by the deletion of *POFUT1* have been implicated in the development of myeloid hyperplasia in adult mice. Moreover, *POFUT1* deficiency provokes a slight decrease in Notch1 and Notch2 expression at the cell surface and abrogates the binding between Notch and its ligand Delta [40]. Additionally, evidence from *Drosophila* suggest that Pofut1 could exert a chaperone activity on the Notch protein. The absence of OFUT1 (Pofut1) leads to the decrease in Notch protein expression on the cell surface, whereas its overexpression increases the binding between Notch and its ligand [41]. Interestingly and consistent with this notion, the absence of OFUT1 leads to the retention of Notch in the endoplasmic reticulum [42]. In human cervical cancer, several reports indicate intracellular localization of the Notch1 protein [42–46]. Moreover, studies on cervical cancer showed that in a normal cervix, Notch-1 is more commonly membrane-localized than in a cancerous cervix [46]. Thus, our results could suggest that under downregulation of the enzyme Pofut1, the protein Notch1 could exhibit a decrease in its O-fucosylation pattern or in its presence at the cell membrane. Alteration of this particular glycogene could promote aberrant Notch signalling [47, 48].

In the second case, higher E6 and E7 expression, the glycogenes *GALNT1* and *C1GALT1C1* are upregulated. Since, GalNAc-T1 and C1GalT1 specific chaperone 1 participate in the Tn antigen O-glycosylation pathway [49, 50], the results suggest that E6 and E7 activity could promote Tn antigen biosynthesis by upregulating the expression of both enzymes. Mucin1 is a membrane-bound protein that participates in intracellular signalling and cell adhesion. The protein is O-glycosylated by several transferases, including GalNAc-T1 and the C1GalT1C1-C1GalT1 complex. Changes in this glycosylation process have been associated with different types of cancer [51]. Specifically, two tumour antigens expressed in carcinomas are Tn and sialyl-Tn; these structures are present in many mucin-type glycoproteins including Muc1 [49–51]. O-glycosylation that leads to Tn synthesis begins with the addition of an N-acetylgalactosamine to a Ser or Thr residue of the protein, catalyzed by GalNT1. Following Tn antigen formation, this new chain can serve as an acceptor for at least three other Golgi glycosyltransferases. The most common modification of the Tn antigen is the formation of the core 1 disaccharide (or T antigen) by the action of C1GalT1 (known as T-synthase), which requires the chaperone C1GalT1C1 (known as COSMC) for its activity. C1GalT1C1 resides in the endoplasmic reticulum and prevents the misfolding, aggregation, and proteasome-dependent degradation of newly synthesized T-synthase. In the absence of functional C1GalT1C1, the newly synthesized T-synthase is inactive and rapidly degraded. Loss of C1GalT1C1 function eliminates T-synthase activity and the consequential Tn antigen expression in several human tumours [50]. In summary, our results suggest an increase in Tn and T antigen synthesis in Mucin1 since the cells displayed an upregulation of both the GalNT1 and C1GalT1C1 proteins. In cervical cancer, Tn and sTn antigens are expressed in invasive squamous cell carcinomas, but not in a normal squamous epithelium [24].

By contrast, GalNT1 can exhibit a mucin-independent function in cancer and be implicated in other pathways, such as EGFR signaling, by increasing EGFR degradation via decreasing of EGFR O-glycosylation [52]. Moreover, GalNT1 can mediate the O-glycosylation of Sonic Hedgehog to promote signal activation in bladder cancer stem cells [53]. In the case of C1GalT1C1, forced expression of the chaperone in colon cancer cell lines increases T antigen expression and enhances cell growth, migration, and invasion, and this phenotype is associated with the increased phosphorylation of ERK and AKT. By contrast, knocking down C1GalT1C1 decreases malignant behaviours and the signalling pathways, suggesting that the chaperone can promote malignant phenotypes of colon cancer cells, mainly via activation of the MEK/ERK and PI3K/Akt signalling pathways [27].

These reports suggest that the glycogenes altered in the microarray assay of HeLa cells with partially silenced E6 and E7 oncogenes, could play important roles in cervical transformation that have not yet been explored.

## Conclusions

Partially silencing of the E6 and E7 HPV oncogenes could be implicated in important glycosylation pathways altered in cervical cancer. In the present study, glycosylation of the Notch receptor and O-glycosylation type mucin were identified. The identified genes implicated in these pathways have not been reported in cervical cancer.

The analysis of glycogenes altered by the HPV oncoproteins E6 and E7 is a valuable tool to identify possible glycogenes implicated in the cervical transformation process and to understand the role of HPV infection in glycosylation changes detected in cervical cancer.

## Abbreviations

CIN: Cervical intraepithelial neoplasia
HPV: Human papillomavirus
polyLacNAc: Polylactosamine
β3GnTs: β1,3-N-acetylglucosaminyl transferases
